# Changes in Subcellular Localization of Host Proteins Induced by Plant Viruses

**DOI:** 10.3390/v13040677

**Published:** 2021-04-15

**Authors:** Rosalba Rodriguez-Peña, Kaoutar El Mounadi, Hernan Garcia-Ruiz

**Affiliations:** 1 Department of Plant Pathology, Nebraska Center for Virology, University of Nebraska-Lincoln, Lincoln, NE 68503, USA; rrodriguezpena2@unl.edu; 2 Department of Biology, Kutztown University of Pennsylvania, Kutztown, PA 19530, USA; elmounadi@kutztown.edu

**Keywords:** antiviral, colocalization, host factors, protein relocalization, proviral, replication proteins, TuMV

## Abstract

Viruses are dependent on host factors at all parts of the infection cycle, such as translation, genome replication, encapsidation, and cell-to-cell and systemic movement. RNA viruses replicate their genome in compartments associated with the endoplasmic reticulum, chloroplasts, and mitochondria or peroxisome membranes. In contrast, DNA viruses replicate in the nucleus. Viral infection causes changes in plant gene expression and in the subcellular localization of some host proteins. These changes may support or inhibit virus accumulation and spread. Here, we review host proteins that change their subcellular localization in the presence of a plant virus. The most frequent change is the movement of host cytoplasmic proteins into the sites of virus replication through interactions with viral proteins, and the protein contributes to essential viral processes. In contrast, only a small number of studies document changes in the subcellular localization of proteins with antiviral activity. Understanding the changes in the subcellular localization of host proteins during plant virus infection provides novel insights into the mechanisms of plant–virus interactions and may help the identification of targets for designing genetic resistance to plant viruses.

## 1. Introduction

The most abundant plant viruses have a genome that is a positive single-strand RNA (Group IV) or a negative single-strand RNA (Group V). Single-strand RNA viruses replicate in compartments or vesicles bound to membranes in the cytoplasm or in subcellular organelles [1]. Plant-infecting DNA viruses, on the other hand, are less numerous. Single-strand DNA (Group II) and reverse-transcribing DNA (Group VII) viruses replicate by forming a minichromosome in the nucleus [2].

Viral RNA is translated into proteins using the cellular machinery. Viral nucleic acids and proteins execute their functions in cooperation with host proteins, RNAs, or other factors such as membranes or lipids [3,4]. These components condition susceptibility, and their absence reduces virus accumulation or movement, and may turn a host into a nonhost. These factors encode loss-of-susceptibility genes, also named susceptibility genes [3,5]. Because the presence and activity of these host components are essential for the virus, the terms cellular factors with proviral activity or proviral host factors are often used in publications [3,6].

The establishment of a viral infection is genetically determined at two sequential phases. Initially, the absence of susceptibility genes results in the lack of infection or reduced virus replication and/or movement [3]. When a plant has the susceptibility genes needed for the initiation of infection, in a second phase, virus accumulation, spread, and disease severity are determined by the balance between plant defense and viral suppression of defense responses [7].

Viral infection induces changes in host gene expression [8,9] resulting in the upregulation of susceptibility genes [10] and activation or downregulation of antiviral defense responses [11,12], and may also lead to up- or downregulation of genes that have no effect on the virus [8,9]. Upregulation of antiviral genes indicates the activation of defense responses by multiple mechanisms including autophagy, RNA decay, or gene silencing [7,13,14]. Antiviral defense is mediated by host factors that target viral proteins or nucleic acids and antagonize key parts of virus replication and/or movement, reducing virus accumulation or limiting the spread of infection within the plant [7,15]. However, to protect themselves, viruses may downregulate expression, suppress activity, or induce degradation of antiviral defense components [16]. The molecular mechanisms and significance of changes in host gene expression during viral infection are still poorly understood.

Viruses divert host proteins from their natural roles to execute essential viral processes such as translation, virus replication, or movement [5,17,18]. Changes in activity are often associated with a change in the subcellular localization of the host protein. A protein is considered to relocalize when, in the presence of a virus, a fraction of the total protein accumulates in a new place in the cell. These changes have been detected and characterized by a combination of approaches such as yeast two hybrid, subcellular fractionation, bimolecular fluorescence complementation, immunofluorescence confocal microscopy, or co-precipitation [19,20,21].

In this review, we present an analysis of publications documenting changes in the subcellular localization of host proteins following viral infection. Results present a profile of the host proteins that change, the experimental approaches used to identify them, their natural and new locations, and their role in favor or against viruses. The profiles advance our understanding of the mechanisms that govern plant–virus interactions and establish the basis for the identification of novel host factors with antiviral activity or that condition virus susceptibility and that can be targeted to generate virus-resistant plants by genetic engineering.

## 2. Profile of Host Proteins

**Figure 1:**
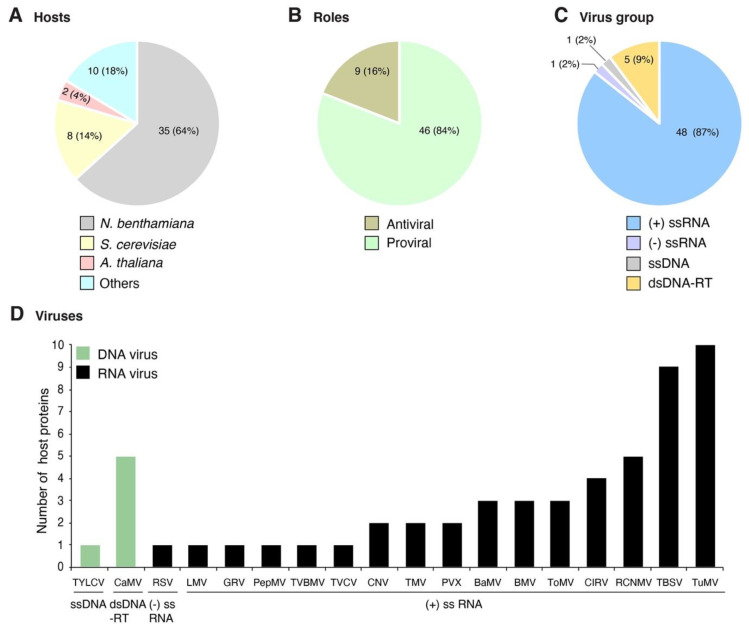
Profile of host proteins that change their subcellular localization during plant virus infection as reported in the literature. Fifty-five combinations of host protein-plant virus were documented in publications. (**A**) Number and proportion of proteins by host species. (**B**) Number and proportion of host proteins with antiviral role or beneficial to the virus. (**C**) Number and proportion of host proteins by virus group. (**D**) Number of host proteins by virus species. Viruses are grouped based on their genome organization.

**Figure 2:**
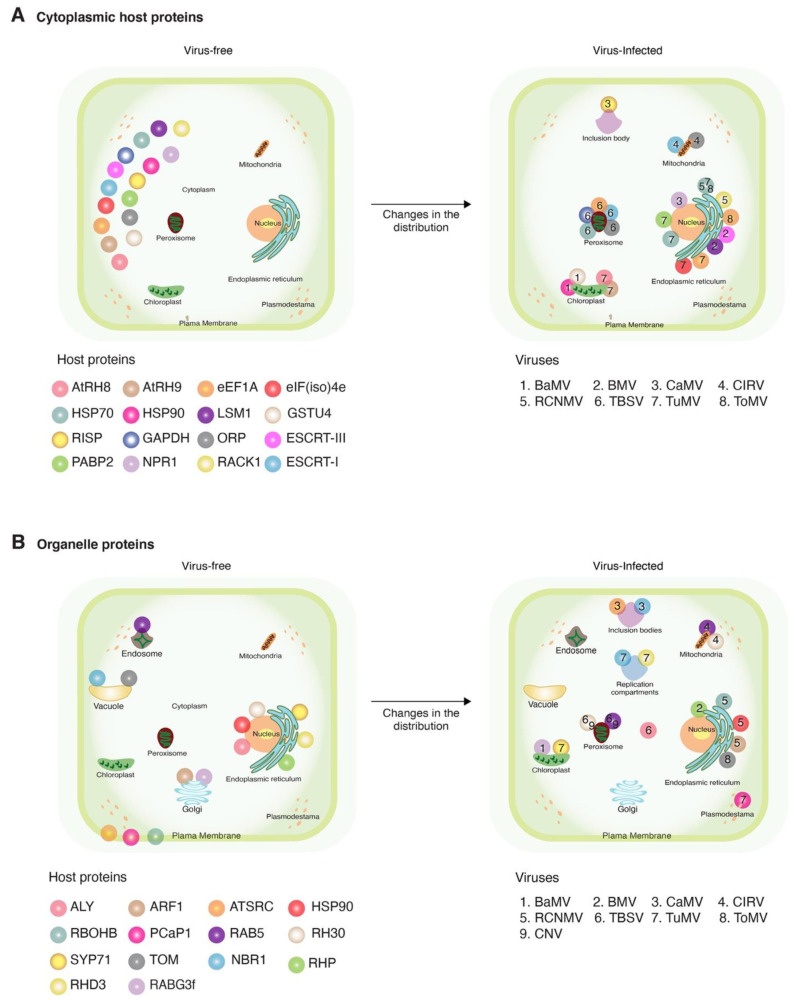
Schematic representation of changes in subcellular localization after viral infection. Representative host proteins and plant viruses that induce relocation in more than two proteins are illustrated. Host proteins are color-coded with spheres. Viruses are indicated by numbers. (**A**) Changes in host cytoplasmic proteins and (**B**) changes in host proteins naturally localized to organelles, and their movement in the presence of a virus.

Plant viruses for which at least one host protein has been reported to change subcellular localization were grouped based on their genome organization. The site of genome replication, name of the replication protein, and movement form were compiled and used as a guide to interpret interaction with and recruitment of host proteins (Table 1). We classified the host proteins based on their natural subcellular localization in the absence of viral infection. Changes in subcellular localization were documented for 55 combinations of host protein and plant virus. After profiling features of these proteins and viruses, several general patterns emerged: (1) 45 of the 55 combinations were identified using model hosts (*Arabidopsis thaliana*, *Nicotiana benthamiana*, or *Saccharomyces cerevisiae*, Figure 1A); (2) the majority (48) were identified using model positive-strand RNA viruses (Figure 1C), particularly brome mosaic virus (BMV), tomato bushy stunt virus (TBSV), and turnip mosaic virus (TuMV) (Figure 1D); (3) in 46 of the 55 combinations, the host protein is beneficial to the virus; (4) host proteins with antiviral roles were less abundant (nine) (Figure 1B); and (5) the most frequent group (30 out of 55) was host proteins that moved from the cytoplasm to the sites of virus replication (Figure 2A and Table 2) through interactions with viral proteins (Table 2). These patterns are heavily influenced by the combination of experimental hosts and viruses used as model systems.

## 3. Cytoplasmic Host Proteins

Of the 55 host proteins that changed their localization during viral infection, 30 were cytoplasmic and moved to the sites of virus replication in the mitochondria, chloroplasts, endoplasmic reticulum (ER), peroxisomes, or nucleus and participated in essential processes such as the formation of the sites of virus replication, stimulation of RNA synthesis, or stability of the RNA-dependent RNA polymerase (Figure 2 and Table 2). Host proteins represented include heat shock proteins, translation factors, and proteins that mediate membrane topology (Table 2). Other proteins include GSTU4 (glutathione transferases), oxysterol-binding protein–related proteins (ORPs), catalase 1, endosomal sorting complexes required for transport (ESCRTs), and like Sm protein 1 (LSm1). Two antiviral proteins (NPR1 and 20S α5) moved from the cytoplasm to the nucleus or virus-induced aggregates. Their new localization was mediated by viral proteins and resulted in the loss of antiviral activity (Table 3). Cytoplasmic proteins with a new distribution in virus-infected cells are discussed below.

### 3.1. Heat Shock Proteins (HSPs)

HSPs are highly conserved in plants and animals. They are chaperones that protect other proteins from degradation and facilitate protein trafficking across membranes. HSPs have several physiological functions in plants, including protection from stress caused by heat, cold, light, heavy metals, salts, and ozone. HSPs are mainly located in the cytosolic part of the cell, and in some cases, in the nucleus, chloroplasts, or ER [88,89]. Several HSPs are chaperones of viral proteins, have essential roles in virus replication [20,21,56], and are recruited to the sites of virus replication (Table 2). Notable examples are discussed below.

HSP70 moves from the cytoplasm to the nucleus during tomato yellow leaf curl virus (TYLCV, single-strand DNA genome) infection [68], and to the ER compartments during TuMV (single positive-strand RNA genome) infection [20]. Downregulation of HSP70 results in the reduced accumulation of TYLCV genomic DNA in infected plants [68]. Both HSP70 and HSP90 localize mainly in the cytoplasm, and upon red clover necrotic mosaic virus (RCNMV, single positive-strand RNA genome) infection, HSP70 and HSP90 were detected in the ER (Table 2). Movement occurred through interactions with p27, a component of the virus replication compartments [31,74]. Without detectable physiological or developmental defects in the plants, the downregulation of HSP70 and HSP90 by virus-induced gene silencing prevented infection by RCNMV, confirming there are susceptibility genes [21,31]. Moreover, during infection with bamboo mosaic virus (BaMV, single positive-strand RNA genome), HSP90 enhances the formation of ribonucleoprotein complexes and facilitates their entry into the chloroplast, thus moving to the chloroplasts and playing an important role in BaMV replication [90]. HSP20 antagonizes the antiviral response by moving into the nucleus through interaction with the RNA-dependent RNA polymerase (RdRp) of rice stripe virus (RSV, single negative-strand RNA genome), blocking the recruitment of viral RNA to stress granules and, thus, blocking the degradation of viral RNA and enhancing viral RNA translation [56].

### 3.2. Endosomal Sorting Complexes Required for Transport (ESCRTs)

ESCRTs are peripheral membrane proteins in plant, mammalian, and yeast cells and play important roles in autophagy, sorting of ubiquitinated receptors, and in cytokinesis. They are also involved in the detachment of membrane vesicles, viral budding, and in the formation of the sites of virus replication [48,58,91]. ESCRT-I and ESCRT-III move from the cytoplasm to the sites of replication formed by TBSV (single positive-strand RNA genome) in the peroxisome and by BMV (single positive-strand RNA genome) in the perinuclear ER (Table 2) [18,48,58]. Their recruitment to the sites of virus replication is mediated by their interaction with TBSV replication protein p33 or BMV replication protein 1a (Table 2). ESCRT proteins are proposed participate in bending membranes to achieve the spherical shape of the compartments that function as sites of replication for both viruses [18,48,58]. Thus, the absence of ESCRT proteins in yeast cells, and their downregulation in plants, resulted in a reduction in the accumulation of BMV and TBSV, respectively [18,48,58].

### 3.3. Translation Factors

Viruses are dependent on the host machinery to translate their RNAs into proteins. Several viruses require eukaryote initiation factors Poly A binding protein2 (PABP2), eukaryote initiation factor eIF (iso)4e, eIF4e, and elongation factor eEF(1A). During viral infection, these proteins move from the cytoplasm to the sites of TuMV replication [5,45,92] (Table 2). Re-initiation supporting proteins (RISPs) move from the cytoplasm to trans-activator viroplasmin (TAV) aggregates [45,93] and are necessary for cauliflower mosaic virus (CaMV) infection [65,66]. Additionally, some translation factors contribute to the cell-to-cell movement and systemic spread of the virus [92]. The role of the initiation, elongation, and re-initiation factors in the sites of virus replication and in virus movement is unclear.

### 3.4. Asp-Glu-Ala-Asp (DEAD)-Box RNA Helicases (RHAs)

DEAD-box RNA helicases (RHAs) are a large family of RNA helicases involved in all steps of RNA metabolism. They are required for transcription, mRNA splicing and translation, RNA modification and transport, ribosome biogenesis, ribonucleoprotein complex assembly, and mRNA degradation [94]. RHAs are also involved in the response to biotic stress and in abiotic stress tolerance [95].

Several RHAs contribute to the translation and replication of viral RNA and change their localization upon viral infection [96,97]. The RNA helicases, AtRH8 and AtRH9, move from the cytoplasm to the sites of TuMV replication in the chloroplast in *A. thaliana* (Table 2). Migration into the chloroplast is mediated by viral proteins. AtRH8 interacts with virus-linked protein VPg [63], while AtRH9 interacts with potyvirus membrane protein 6K2 [64]. In mutant plants lacking either AtRH8 or AtRH9, accumulation of TuMV is reduced [63,64].

### 3.5. Glyceraldehyde 3-Phosphate Dehydrogenase (GAPDH)

GAPDH is a catalytic enzyme involved in glycolysis and mRNA binding. After infection with TBSV, GAPDH moves from the cytoplasm to the peroxisomal membrane and into the sites of TBSV replication. GAPDH is responsible for maintaining the ratio between positive- and negative-strand RNA genomes. Downregulation of GAPDH in *N. benthamiana* plants caused a fourfold reduction in the accumulation of TBSV and tobacco mosaic virus (TMV, single positive-strand RNA genome), pointing to a required role of this protein in both viruses [59]. In contrast, GAPDH has an antiviral role against BaMV and its satellite (satBaMV) as its downregulation in *N. benthamiana* plants resulted in an increase in BaMV and satBaMV RNA accumulation, while its overexpression reduced the accumulation of BaMV and satBaMV. The subcellular localization of GAPDH does not change during infection with BaMV or satBaMV [98].

### 3.6. Glutathione Transferase U4 (GSTU4)

GSTU4 belongs to the family of plant glutathione transferases (GSTs), which catalyze the reduction of hydroperoxides formed during oxidative stress, participate in ultraviolet-inducible cell signaling pathways, and in the regulation of apoptosis [99]. During infection with BaMV, GSTU4 is upregulated, interacts with the 3′ untranslated region (UTR) in BaMV genomic RNA, moves from the cytoplasm to the sites of virus replication in the chloroplasts, and enhances the synthesis of negative-strand RNA. Downregulation of GSTU4 caused a reduction in the accumulation of BaMV and potato virus X (PVX, single positive-strand RNA genome) but not in the accumulation of cucumber mosaic virus (CMV, single positive-strand RNA genome) or TMV [47]. In soybean, GSTU10-10 is induced in response to the systemic infection of the plants by soybean mosaic virus (SMV, single positive-strand RNA genome), but no subcellular localization data were reported [100]. Thus, GSTs may have a role in virus susceptibility, mediated by their ability to reduce oxidative stress, which supports viral replication.

### 3.7. Other Cytoplasmic Proteins

Other cytoplasmic proteins with altered localization during viral infection include oxysterol-binding protein–related proteins (ORPs), a yeast RNA-binding protein (LSm1), receptor for activated C kinase 1 (RACK1), and a proteasome protein (20S α5). ORPs are lipid transfer proteins that participate in vesicular trafficking, lipid metabolism and signaling, non-vesicular sterol transfer, directional sterol transport, and other processes [101]. ORPs facilitate the redistribution of sterols and enhance membrane folding during the formation of the replication sites in the peroxisomes by TBSV, and in the mitochondrial membrane by carnation Italian ringspot virus (CIRV, single positive-strand RNA genome) (Table 2). Deletion of ORPs lowers the efficiency of the viral replicase assembly and activity and resulted in a major reduction in virus accumulation [52].

LSm1 is a yeast protein related to the core small nuclear ribonucleoprotein particle (snRNP). LSm1 is involved in BMV RNA de-capping and translation. LSm1 protein is recruited from the cytoplasm to the ER by replication protein 1a and conditions susceptibility to BMV [50].

RACK1 is a cytoplasmic protein involved in the regulation of several plant processes including development, hormone response, and environmental stress response [102]. It is also involved in the innate immune response against fungal and bacterial pathogens [103,104,105]. During RCNMV infection of *N. benthamiana*, RACK1 interacts with viral replication protein p27 and moves from the cytoplasm to the sites of replication in the ER (Table 2). RACK1 is essential for the p27-mediated induction of reactive oxygen species (ROS) bursts that enhances virus replication. Downregulation of RACK1 resulted in the reduced accumulation of RCNMV RNA [55].

The 20S α5 is a subunit of the ubiquitin–proteasome system with RNAse activity and is involved in the degradation of viral RNA [83,84]. After infection with lettuce mosaic virus (LMV, single positive-strand RNA genome), the 20S α5 colocalizes with HC-Pro in cytoplasmic aggregates (Table 3). Interaction with HC-Pro and movement into cytoplasmic aggregates blocks the RNAse activity of 20S α5, protecting the virus from degradation. Consistent with its antiviral role, downregulation of 20S α5 enhances LMV accumulation [84].

## 4. Endosomal Proteins

Rab GTPases are central regulators of vesicle budding, movement, and fusion [106]. Endosomal protein Rab5-small guanosine triphosphatase (RAB5-GTPase) regulates endosome biogenesis and homotypic and heterotypic fusions [107]. During infection with TBSV and CNV (Table 2), RAB5-GTPase moves to the sites of virus replication in the peroxisomes, or in the mitochondria during CIRV infection [69]. RAB5-GTPase increases the amount of endosomal phospholipid phosphatidylethanolamine needed to form the sites of virus replication and to establish robust virus replication [69].

## 5. Endoplasmic Reticulum Proteins

Several RNA viruses form their sites of replication in endoplasmic reticulum (ER)-bound membranes [108], and several ER proteins move into the sites of virus replication formed in other subcellular organelles (Figure 2B). Representative groups are discussed below (Table 2).

### 5.1. Soluble N-Ethyl Maleimide Sensitive Factor Adaptor Protein Receptors (SNAREs)

SNAREs belong to the syntaxin family of proteins that mediate membrane fusion between transport replication compartments and their target membranes [109]. The potyviral 6K2 protein induces the formation of ER-derived complexes that subsequently translocate to the chloroplasts, where potyviral replication occurs [71]. *A. thaliana* SYP71 is a SNARE protein located both in the ER and in the plasma membrane [110], is essential for TuMV replication, and contributes to the movement of replication compartments from the ER to the chloroplast (Table 2). Downregulation of SYP71 inhibits TuMV infection [71].

### 5.2. Reticulon Homology Domain Proteins (RHPs)

RHPs are a family of membrane-shaping proteins that induce and stabilize positively curved peripheral ER membranes and are involved in apoptosis, cell division, and intracellular trafficking [111]. RHPs normally localize to the peripheral ER. During BMV infection, they interact with replication protein 1a, move to the perinuclear ER, and are incorporated into BMV sites of replication. RHPs are essential for the formation of BMV replication compartments. Mutant plants lacking RHPs have a reduced number of replication compartments and an 80% of reduction in viral replication compared to the wild-type plants [70].

### 5.3. Root Hair Defective 3 (RHD3)

RHD3 is a member of the dynamin-like atlastin GTPase family of proteins. It plays a vital role in generating of the interconnected tubular ER network, is required for Golgi distribution and motility in plant cells, and is essential for plant development [112]. RHD3 is essential for the formation, maturation, and intracellular movement of TuMV replication compartments. Interaction with TuMV 6K2 protein moves RHD3 from the ER to the TuMV replication compartments (Table 2). In mutant plants lacking RHD3, replication and systemic movement of TuMV are reduced [28].

## 6. Golgi Apparatus Proteins

Rab guanosine triphosphatase 3f (RABG3f) belongs to the family of Rab GTPases that regulate the intracellular trafficking between organelles [113]. RABG3f is located in the Golgi apparatus and moves to the chloroplast during BaMV infection (Table 2), participates in the formation of the sites of replication, and is required for the efficient infection of in *N. benthamiana* by BaMV. Downregulation of NbRABG3f reduces the accumulation of BaMV [72].

ADP-ribosylation factor (ARF1) 1 is a Golgi body GTPase [114]. During infection with RCNMV, ARF1 interacts with viral protein p27 and is moved into the sites of virus replication on the ER membrane (Table 2). Inhibition of ARF1 activity causes a reduction in RCNMV RNA accumulation [74].

## 7. Plasma Membrane Proteins

Viruses move from cell to cell through plasmodesmata using specialized viral movement proteins and host proteins [115]. Potyviral replication compartments are transported to the plasmodesmata throughout the ER–Golgi secretory pathway and the actomyosin motility system [40]. The phosphatidylinositol phosphates Ca-binding protein (PCaP1) and the developmentally regulated protein (DREPP) are plasma membrane proteins that mediate the cell-to-cell movement of TuMV and tobacco vein banding mosaic virus (TVBMV, single positive-strand RNA genome) respectively, by interacting with P3N-PIPO and CI (cylindric inclusion) proteins (Table 2). The interaction between PCaP1 or DREPP and P3N-PIPO or CI and their relocalization to the plasmodesmata are required for efficient virus replication and local and systemic movement [40,77]. CaMV (single-strand DNA genome) moves through the plasmodesmata as a virion. Cytoplasmic and nuclear inclusion bodies necessary for virion assembly are formed by viral protein P6. AtSRC2.2, a C2 calcium-dependent membrane-targeting protein, is part of the inclusion bodies and has been implicated in CaMV movement [75].

The respiratory burst oxidase homolog (RBOHB) is a plasma membrane protein in the family of plant NADPH oxidases and plays an essential role in ROS production and signaling [116]. During infection of *N. benthamiana* by RCNMV, RBOHB moves to the perinuclear ER-bound sites of virus replication (Table 2) through interactions with replication protein p27 and is required for robust viral RNA replication [19]. In contrast, during infection with TMV, RBOHB-induced ROS burst has an antiviral effect, with no protein relocalization reported [117,118].

## 8. Nuclear Proteins

DNA viruses replicate in the nucleus [2]. RNA viruses do not enter the nucleus. However, some RNA viruses need nuclear host factors to replicate or move. Fibrillarin is a major nucleolar protein that forms part of Cajal bodies, is a core component of small nucleolar ribonucleoprotein particles, and is required for rRNA processing. Similar to all other umbraviruses, groundnut rosette virus (GRV, single positive-strand RNA genome) does not encode for a coat protein and does not form virions. The lack of a coat protein is compensated by open reading frame 3 (ORF3), and GRV moves as ribonucleoprotein particles. In infected plants, ORF3 cycles from the cytoplasm to the nucleus passing through Cajal bodies and back into the cytoplasm. This movement is dependent on interactions between ORF3 and fibrillarin and is required for the formation of ribonucleoprotein particles. Downregulation of fibrillarin through virus-induced gene silencing in *N. benthamiana* did not affect virus replication or cell-to-cell movement. However, in the absence of fibrillarin ORF3 remained in the Cajal bodies, failed to fuse with the nucleolus, and prevented systemic movement of GRV [27].

Allies of LEF-1 and AML-1 (ALY proteins) belong to a family of nuclear polypeptides involved in mRNA export from the nucleus to the cytosol, in the regulation of plant immunity, in controlling the aperture of stomata, and plant growth and development [119].

During infection by TBSV, two *A. thaliana* ALYs (AtALY2 and AtALY4) and an *N. benthamiana* ALY protein (NbALY617) move from the nucleus to the cytoplasm (Table 2) by interacting with P19, a strong silencing suppressor of cytoplasmic distribution that interferes with antiviral gene silencing [79]. The biological significance of this relocation and interaction remains to be determined. In contrast, *A. thaliana* proteins AtALY1 and AtALY3 and *N. benthamiana* proteins NbALY615 and NbALY1693 inhibit the silencing suppression activity of P19 (Table 3). This effect is mediated by the sequestration of P19 in the nucleus by nuclear ALY proteins via an RNA-binding motif [120].

Nuclear DEAD-box RNA helicase RH30 interacts with TBSV replication proteins p33 and p92^pol^ and moves from the nucleus to TBSV sites of replication in the peroxisome (Table 3). RH30 inhibits the formation of replication compartments, the recruitment of genomic positive-strand RNA into replication, and RNA synthesis. The antiviral effect has been shown against TBSV, CNV, CIRV, RCNMV, and TMV [86].

## 9. Vacuolar Proteins

Tobamovirus multiplication 1 (TOM1) and TOM3 (Table 2) are vacuolar membrane proteins required for the replication of tobamoviruses. Both interact with the helicase-like domain of replication proteins 130K and 180K in tomato mosaic virus (ToMV, single positive-strand RNA genome) and participate in the formation and anchoring of replication compartments to the ER [80,81].

Autophagy is a conserved mechanism of protein degradation involved in cell homeostasis. Autophagy may benefit the virus or have antiviral roles [121,122]. Selective autophagy substrate, NBR1, is a cargo receptor protein that suppresses TuMV and CaMV infection by targeting the silencing suppressor HC-Pro and the structural capsid protein P4, respectively. To create an environment favorable to replication and movement, TuMV antagonizes NBR1-dependent autophagy by a mechanism that is dependent on the viral proteins VPg and 6K2 (Table 3). NBR1 is normally found in the autophagosome, cytoplasm, and vacuoles. During TuMV infection, NBR1 moves to virus replication compartments [123]. NBR1 mediates the degradation of TuMV HC-Pro. In turn, TuMV VPg and 6K2 proteins counteract NBR1 antiviral effects [87]. However, the role of NBR1 in plant–TuMV interactions is more complex. During TuMV infection of *Brassica napus*, large amounts of secondary siRNAs are formed from the NBR1 mRNA, which in turn direct cleavage and downregulation of NBR1 mRNA, actin depolymerization factor (ADF), and other transcripts [124].

## 10. Conclusions

Host proteins needed by the virus may be redirected to subcellular compartments where they contribute to essential viral processes, such forming sites of viral replication, RNA synthesis, or virus movement (Table 2 and Figure 2). In the opposite direction, host proteins with antiviral roles may be forced to move away from their natural subcellular localization. Changes in subcellular localization of host proteins are mediated by direct or indirect interactions with viral proteins or RNA (Table 2). However, it is not clear whether accumulation in a new location results from the movement of existing host proteins or from newly synthesized ones.

Changes in the subcellular localization of host proteins have been identified and characterized mainly using experimental hosts *N. benthamiana*, *A. thaliana,* and *S. cerevisiae* (Figure 1A). The most frequent change is cytoplasmic proteins redirected to virus replication compartments formed in the ER, peroxisome, chloroplast, mitochondria, and nucleus (Figure 1 and Figure 2). In contrast, the number of studies documenting changes in subcellular localization of antiviral proteins is significantly (fivefold) lower (Figure 1B and Table 3). These antiviral proteins block the formation of replication compartments or degrade viral proteins. Subcellular relocalization was needed to perform antiviral activities. Interestingly, in some cases, antiviral activity is inhibited by virus-induced changes in localization (Table 3).

Mutational inactivation or downregulation of proteins that participate in essential viral processes results in lack of infection or reduction of viral RNA accumulation [5,46]. However, genetic changes in the host may cause developmental abnormalities, reduced fitness, or altered physiology, masking the real effect or indirectly affecting virus replication or movement. HSP70, RABG3f, GSTU4, RACK1, GAPDH, AtRH8, and AtRH9 were downregulated by silencing or mutationally inactivated. The source paper explicitly indicated that plants did not show detectable physiological or severe developmental defects compared to the wild-type plants. RACK1-knockdown plants had narrow leaves, categorized as a minor physiological defect [55]. Other publications did not provide information on the phenotype of mutant plants.

The dependence of viruses on host resources at all steps of the infection process puts evolutionary pressure both on the virus and on the host. Virus–host co-evolution might favor interactions that increase both host and virus fitness rather than decreasing fitness of either the host or the virus. Accordingly, it might be to the benefit of viruses to interact with existing processes at their natural location in a non-interfering manner rather than through mechanisms that recruit host proteins away from their natural sites and normal functions, which may potentially lead to disease. All documented cases of changes in subcellular localization (Table 2 and Table 3) come from viruses that are pathogenic. Currently, no information is available on the changes, or lack thereof, of host proteins for virus–plant combinations in which the interaction does not lead to disease.

Recruitment of cytoplasmic proteins to the sites of virus replication in membrane-bound compartments through mechanisms that include direct protein–protein interactions between viral and host proteins and indirectly through membranes, other proteins, or RNA (Table 2, Figure 2) imposes selection pressure on viral proteins to maintain the ability to interact with host proteins. Viral proteins are usually multifunctional. In addition to interacting with other viral proteins or RNA, efficient interaction with host proteins must be maintained. The host might be genetically uniform or diverse. These constraints exert selection pressure on viral proteins to maintain functionality and be able to interact with the cognate host proteins. These observations suggest that interactions between viral and host proteins that are essential at any part of the infection process are also important determinants of virus evolution and host adaptation. Viral proteins might respond to selection pressure by developing a perfect sequence and structure that is functional and able to interact with host proteins that are genetically diverse. Alternatively, viral proteins might respond to selection pressure by incorporating mutationally robust areas coding for structurally flexible domains capable of interacting with host proteins that are genetically diverse. Viruses might be generalists with a wide host range or specialists with a narrow host range. Through co-evolution, in response to selection pressure from the virus, plants might generate diversity in their alleles and/or by incorporating redundant alleles.

This model is illustrated by translation initiation factors and potyviruses. The translation initiation factors, eEF1A and eIF(iso)4e, participate in TuMV viral RNA translation and formation of replication compartments through interactions with VPg [5,125]. Although eIF(iso)4e is necessary for potyvirus infection, it is dispensable for normal plant growth and development [5]. In addition to interacting with eIF(iso)4e, VPg must recognize and interact with TuMV genomic RNA, the RNA-dependent RNA polymerase, and protein 6K1 [5,125]. In connection with its multifunctionality and ability to interact with multiple partners, potyviral VPg is structurally disordered and mutationally robust [126]. Translation initiation factors eIF4E are a multigene family. Ten allelic variants of eIF4 were detected in natural pepper populations with varying levels of susceptibility to potyviruses [127]. Accessions resistant to potyvirus infection encoded mutations in eIF4 that disrupted interaction with VPg [127], consistent with the essential role of both VPg and eIF4 in potyvirus–plant interactions and with the model that plants respond to selection pressure by accumulating diversity to buffer the effect of viral infection.

Changes in subcellular localization of host proteins documented to date and summarized here represent bonafide events associated with basic mechanisms of plant–virus interactions. However, these patterns are heavily influenced by the combinations of model hosts and viruses used to develop model experimental systems to identify host factors that affect virus replication (Figure 1A). A large fraction of the host proteins documented were identified using only three viruses BMV, TBSV, and TuMV (single positive-strand RNA, Figure 1D). Reasons for this bias mainly include the availability of infectious clones; well-characterized experimental systems to study virus replication, gene expression, and function; and genetically tractable heterologous hosts in combination with well-developed biochemical approaches for protein expression, purification, and localization [28,128,129]. Overexpression of viral proteins in non-natural hosts is a powerful tool to identify potential host proteins that play important roles during infection. It is possible that some of the resulting relocalization may be an artifact of overexpression of individual viral proteins in a heterologous system. However, genetic analyses, virus-induced gene silencing, chemical treatments, or a combination of approaches have been used to validate the requirement of particular host proteins, ruling out the possibility of an experimental artifact [21,28,68]. Furthermore, basic information about factors required for viral infection identified in heterologous hosts has been used to engineer resistance in crops. Based on the observation that eukaryote translation initiation factors are susceptibility genes to potyviruses [5], through interaction with potyviral VPg [28], CRISPR-Cas9 was used in tomato to engineer resistance to pepper mottle virus by editing eIF4E1 [130].

New approaches are needed for the genome-wide identification of factors required for the virus or with antiviral activities in natural hosts. Although these approaches are likely to be developed using model experimental systems, it would be of immense benefit to implement them in staple crops. Alternatively, or in addition, these approaches could be directed to particular diseases to which natural resistance is not readily available, such as maize lethal necrosis [131].

## Figures and Tables

**Table 1:** Site of replication and movement form of plant viruses for which at least one host protein has been reported to change subcellular localization.

Virus	RdRp	Site of Replication	Intracellular Movement of the Replication Compartments	Cell-To-Cell Movement Form	Reference
**Group II: Single-strand DNA**					
TYLCV	Rep protein	Nucleus	From nucleus to plasmodesmata	Minichromosome	[22]
**Group IV: Single positive-strand RNA**					
BaMV	155 kDa	Chloroplast	From chloroplast to plasmodesmata	Virions or ribonucleoprotein particles	[23]
BMV	2a	Endoplasmic reticulum	Non-motile	Virions or ribonucleoprotein particles	[24]
CIRV	p36	Mitochondria	Non-motile	Ribonucleoprotein particles	[25]
CNV	p33	Peroxisome	Non-motile	Ribonucleoprotein particles	[26]
GRV	RdRp	Cytoplasm	NA	Ribonucleoprotein particles	[27]
LMV	NIb	Endoplasmic reticulum	From ER to plasmodesmata	Replication vesicles	[28]
PepMV	164 kDa	Cytoplasm (membrane association with ER is unclear)	From cytoplasm to plasmodesmata	Ribonucleoprotein particles	[29]
PVX	RdRp	Endoplasmic reticulum	NA	Virions or ribonucleoprotein particles	[30]
RCNMV	p27 and p88	Endoplasmic reticulum	From ER to plasmodesmata	Virions	[31,32,33]
TVCV	RdRp	Endoplasmic reticulum	From ER to plasmodesmata	Virions or ribonucleoprotein particles	[34]
TBSV	p92^pol^	Peroxisomes	Non-motile	Ribonucleoprotein particles	[35,36]
TMV	RdRp	Endoplasmic reticulum	From ER to plasmodesmata	Replication complexes or ribonucleoprotein particles	[37]
ToMV	130K and 180K	Endoplasmic reticulum	From ER to plasmodesmata	Virions or ribonucleoprotein particles	[38]
TuMV	NIb	ER and chloroplasts	From ER to chloroplast and/or to Golgi apparatus and to plasmodesmata	Replication vesicles	[28]
TVBMV	NIb	Chloroplasts	From ER to chloroplast and/or to Golgi apparatus and to plasmodesmata	Replication vesicles	[39,40]
**Group V: Single negative-strand RNA**					
RSV	337 kDa	Cytoplasm (membrane association is unknown)	From ER to Golgi to plasmodesmata	Virion–protein complexes	[41,42,43]
**Group VII: Double-strand DNA-RT**					
CaMV	Rep protein	Nucleus	From nucleus to ER and/or directly to plasmodesmata	Virions	[44]

**Viruses**: bamboo mosaic virus (BaMV), brome mosaic virus (BMV), cauliflower mosaic virus (CaMV), carnation Italian ringspot virus (CIRV), cucumber necrosis virus (CNV), groundnut rosette virus (GRV), lettuce mosaic virus (LMV), pepino mosaic virus (PepMV), potato virus X (PVX), red clover necrotic mosaic virus (RCNMV), rice stripe virus (RSV), turnip vein-clearing virus (TVCV), tobacco mosaic virus (TMV), tomato bushy stunt virus (TBSV), tomato mosaic virus (ToMV), turnip mosaic virus (TuMV), tobacco vein banding mosaic virus (TVBMV), tomato yellow leaf curl virus (TYLCV). **Viral proteins**. NIb: nuclear inclusion protein b, the RNA-dependent RNA polymerase in potyviruses; RdRp: RNA-dependent RNA polymerase; NA: information not available; ER: endoplasmic reticulum.

**Table 2:** Host proteins that participate in essential viral processes and that change subcellular localization during viral infection. Host proteins are organized based on their natural distribution in the absence of virus.

Virus	Viral Protein or RNA	Host Protein	Host	Movement of Host Protein into	Role	Initial Detection	Mechanism of Interaction	Experimental System for Detecting of New Localization Sites *	Method of Observation: Time	Reference
**Cytoplasmic proteins**										
CaMV	TAV	RISP	*Arabidopsis thaliana*	Inclusion bodies (cytoplasmic and nuclear)	Stimulates translation re-initiation	Yeast two hybrid	Protein–protein	*Brassica rapa* leaves	Immunofluorescence and confocal microscopy: 15 dpi	[45]
BaMV	155 kDa and 3′ UTR	HSP90	*Nicotiana benthamiana*	Chloroplast	Formation of replication compartments	Partially purified replicase	Protein–protein and RNA–protein	*Saccharomyces cerevisiae* and *Escherichia coli*	Yeast two hybrid, GST-pull down	[46]
	3′ UTR	NbGSTU4	*N. benthamiana*	Chloroplast	Binds to the 3′ UTR and stimulates negative-strand RNA synthesis	Partially purified replicase	RNA–protein	*E. coli*	UV crosslink	[47]
BMV	1a	ESCRT- III	*S. cerevisiae*	Perinuclear ER	Formation of replication compartments	Yeast genetic analysis	Protein–protein	*S. cerevisiae*	Immunofluorescence and confocal microscopy: 48 h	[48]
	1a and 2b	LSM1	*S. cerevisiae*	ER	Promotes viral RNA translation	Yeast mutagenesis	Protein–protein	*S. cerevisiae*	Immunofluorescence and confocal microscopy: 48 h	[49,50]
CIRV	p36	ESCRT-I	*N. benthamiana*	Mitochondria	Formation of replication compartments	Split ubiquitin assay	Protein–protein	*S. cerevisiae*	Immunofluorescence and confocal microscopy: 15-45 min	[51]
	p36	ORP	*N. benthamiana* and *S. cerevisiae*	Mitochondria and ER	Formation of replication compartments	Yeast two hybrid	Protein–protein	*N. benthamiana* leaves	BiFC: 48 h	[52]
PepMV	p26	Catalase 1	*Solanum lycopersicum*	Cytoplasm and nucleus	Antagonist to antiviral response	Yeast two hybrid	Protein–protein	*N. benthamiana* leaves	BiFC, immunolabeling, and electron microscopy: 3–4 dpi	[53]
PVX	TGB12K	TIP	*Nicotiana tabacum*	Peripheral bodies	Regulates plasmodesmata opening	Yeast two hybrid	Protein–protein	*N. benthamiana* leaves	Confocal microscopy: 3 dpi	[54]
RCNMV	p27	HSP70	*N. benthamiana*	ER	Formation of replication compartments	Affinity purification	Protein–protein	*N. benthamiana* leaves	Confocal microscopy: 3 dpi	[21]
	p27	NbRACK1	*N. benthamiana*	ER-derived aggregates	Increases ROS to benefit the virus	Co-immunoprecipitation	Protein–protein	*N. benthamiana* leaves	BiFC: 4 dpi	[55]
RSV	337 kDa	HSP20	*N. benthamiana* and *Oryza sativa*	Nucleus	Antagonist to antiviral response	Yeast two hybrid	Protein–protein	*N. benthamiana* leaves	BiFC: 48 h	[56]
TBSV	p33	eEF1A	*S. cerevisiae*	Peroxisomal membrane	Stabilization of p33	Purified replicase proteomics	Protein–protein	*S. cerevisiae*	Co-purification	[35,57]
	p33	ESCRT-I	*N. benthamiana*	Peroxisomal membrane	Formation of replication compartments	Split ubiquitin assay	Protein–protein	*S. cerevisiae*	Confocal microscopy: 15–45 min	[58]
	p33	GAPDH	*N. benthamiana* and *S. cerevisiae*	Peroxisomal membrane	Viral genomic RNA synthesis	Purified replicase proteomics	Indirect: mediated by p92pol	*S. cerevisiae*	Confocal microscopy: 16 h	[59]
	p33 and p92^pol^	HSP70	*S. cerevisiae*	Peroxisomal membrane	Formation of replication compartments	Reconstitution assay	Protein–protein	*S. cerevisiae*	Confocal microscopy: 16 and 24 h	[60,61]
	p33	ORP	*S. cerevisiae*	Peroxisome and ER	Formation of replication compartments	Affinity purification	Protein–protein	*S. cerevisiae* and *N. benthamiana* leaves	BiFC: 2 dpi	[52]
TMV	RdRp and3′ UTR	eEF1A	*N. tabacum*	Replication compartment	Formation of replication compartments and cell-to-cell movement	Pull-down assay	Protein–protein	*N. tabacum*	Immunoprecipitation: 4 dpi	[62]
TuMV	VPg	AtRH8	*Prunus persica* and *A. thaliana*	Chloroplast membrane	Formation of replication compartments	Yeast two hybrid	Protein–protein	*N. benthamiana* leaves	BiFC: 2 and 10 dpi	[63]
	6K2	AtRH9	*A. thaliana*	Chloroplast membrane	Formation of replication compartments	Confocal microcopy	Protein–protein	*N. benthamiana* leaves	Confocal microscopy: 72 h	[64]
	VPg and NIb	eEF1A	*A. thaliana*	ER-derived replication compartments	Viral RNA translation, formation of replication compartments	Tandem affinity purification	Protein–protein	*N. benthamiana* leaves	Immunofluorescence and confocal microscopy: 4–5 dpi	[65]
	VPg	eIF(iso)4e	*A. thaliana*	ER and chloroplasts	Viral RNA translation, formation of replication compartments	Pull-down assay	Protein–protein	*N. benthamiana* leaves	Immunofluorescence and confocal microscopy: 2–4 dpi	[65]
TuMV	NIb	HSP70	*A. thaliana*	Nucleus and replication compartments in the ER	Formation of replication compartments, regulation of NIb activity	Tandem affinity purification	Indirect: mediated by RdRp	*N. benthamiana* leaves	Confocal microscopy: 2–4 dpi	[20]
	VPg	PABP2	*Brassica perviridis*	Nucleus and ER	Formation of replication compartments	Subcellular fractionation	Protein–protein	*N. benthamiana* leaves	Confocal microscopy: 4–5 dpi	[66]
ToMV	130K and 180K	eEF1A	*N. tabacum*	ER membranes	Viral RNA translation, formation of replication compartments	Subcellular fractionation	Protein–protein	Transgenic *N. tabacum* BY-2 protoplast	Affinity purification	[67]
	130K and 180K	HSP70	*N. tabacum*	ER membranes	Formation of replication compartments	Subcellular fractionation	Protein–protein	Transgenic *N. tabacum* BY-2 protoplast	Affinity purification	[67]
TYLCV	CP	HSP70	*S. lycopersicum*	Cytoplasm and nucleus aggregates	Movement of virions	Subcellular fractionation	Protein–protein	*S. lycopersicum* leaves	Immunodetection and confocal microscopy: 28 or 49 dpi	[68]
**Endosomal proteins**										
CIRV	p36	RAB5-GTPase	*A. thaliana*	Mitochondria	Formation of replication compartments	Yeast two hybrid	Protein–protein	*N. benthamiana* leaves	BiFC: 2 dpi	[69]
CNV	p33	RAB5-GTPase	*A. thaliana*	Peroxisome	Formation of replication compartments	Yeast two hybrid	Protein–protein	*N. benthamiana* leaves	BiFC: 2 dpi	[69]
TBSV	p33	RAB5-GTPase	*A. thaliana*	Peroxisome	Formation of replication compartments	Yeast two hybrid	Protein–protein	*N. benthamiana* leaves	BiFC: 2 dpi	[69]
**Endoplasmic reticulum proteins**										
BMV	1a	RHP	*S. cerevisiae*	Perinuclear ER membrane	Formation of replication compartments	Immunoprecipitation	Protein–protein	*S. cerevisiae*	Co-Ip and confocal microscopy: 12 dpi	[70]
TuMV	6K2	SNARE -SYP71	*A. thaliana*	Chloroplast	Fusion replication compartments in chloroplast	Confocal microscopy	Indirect: mediated by Vap27-1	*N. benthamiana* leaves	Confocal microscopy: 48 h	[71]
	6K2	RHD3	*A. thaliana*	Replication compartments	Maturation of replication compartments	Yeast two hybrid	Protein–protein	*N. tabacum* leaves	Confocal microscopy: 7 dpi	[28]
**Golgi apparatus proteins**										
BaMV	NA	RABG3f	*N. benthamiana*	Replication compartments	Formation and movement of replication compartments	Immunofluorescence	Unknown	*N. benthamiana* leaves	Confocal microscopy: 5 dpi	[72]
CaMV	MP	µA-adaptin	*A. thaliana*	Plasma membrane	MP trafficking	GST pull-down	Protein–protein	*Escherichia coli* and *A. thaliana*	GST-pull down	[73]
RCNMV	p27	ARF1	*N. benthamiana* and *N. tabacum*	ER	Formation of replication compartments	Affinity purification	Protein–protein	*N. tabacum* protoplast	Confocal microscopy: 16 h	[74]
**Plasma membrane proteins**										
CaMV	p6	AtSRC2.2	*A. thaliana*	Inclusion bodies (cytoplasmic and nuclear)	Cell-to-cell movement	Yeast two hybrid	Protein–protein	*N. benthamiana* leaves	Co-immunoprecipitation and confocal microscopy: 3 dpi	[75]
RCNMV	p27	RBOHB	*N. benthamiana*	Perinuclear ER-derived aggregates	ROS synthesis	Immunoprecipitation	Protein–protein	*N. benthamiana* leaves	Confocal microscopy andBiFC : 4 dpi	[76]
TVBMV	P3N-PIPO and CI	DREPP	*N. benthamiana*	Plasmodesmata	Cell-to-cell movement	Yeast two hybrid	Protein–protein	*N. benthamiana* leaves	BiFC: 2 and 5 dpi	[40]
**Plasma membrane proteins**										
TVCV	MP	SYTA	*A. thaliana*	Plasmodesmata	Alters plasmodesmata permeability	Confocal microscopy	Protein–protein	*N. benthamiana* leaves	Confocal microscopy and BiFC	[34]
TuMV	P3N-PIPO	PCaP1	*N. benthamiana*	Plasmodesmata	Cell-to-cell movement	Yeast two hybrid	Protein–protein	*N. benthamiana* leaves	BiFC: 38 h	[77]
**Nuclear proteins**										
GRV	ORF3	Fibrillarin	*N. benthamiana* and *A. thaliana*	Cytoplasm	Systemic movement	Affinity purification and chromatography	Protein–protein	*N. benthamiana* leaves and *E. coli*	Far Western blotting	[27]
RCNMV	p27	HSP90	*N. benthamiana*	ER	Formation of replication compartments	Partially purified replicase	Protein–protein	*N. benthamiana* leaves	BiFC: 3 and 4 dpi	[21]
TMV	MP	NTH201	*N. tabacum*	Cytoplasm and plasmodesmata	Enhances replication compartment formation	Confocal microscopy	Indirect	*N. benthamiana* leaves	Confocal microscopy: 24 h	[78]
TBSV	p19	ALY	*N. benthamiana* and *A. thaliana*	Cytoplasm	Co-factor	Yeast two hybrid	Protein–protein	*N. benthamiana* leaves	Confocal microscopy: 3 dpi	[79]
**Vacuolar proteins**										
ToMV	130K and 180K	TOM1 TOM3	*A. thaliana* and *N. tabacum*	ER	Formation and anchoring of replication compartments	Membrane flotation	Protein–protein	*S. cerevisiae* and *N. tabacum* leaves	Yeast two hybrid and subcellular fractionation at 2 dpi	[80,81]

* Experimental plants are wild type unless noted. BiFC: bimolecular fluorescence complementation; Co-Ip: co-immunoprecipitation; dpi: days post inoculation, agroinfiltration, or induction; NA: information not available.

**Table 3:** Host proteins with antiviral activity and that change subcellular localization in the presence of a plant virus. Host proteins are organized in blocks based on their natural distribution in the absence of virus.

Virus	Viral Protein or RNA	Host Protein	Host	Movement of Host Protein into	Role	Initial Detection Experiment	Mechanism of Interaction	Experimental System for Detecting New Localization Sites *	Method of Observation: Time	Reference
**Cytoplasmic proteins**										
CaMV	P6	NBR1	*A. thaliana*	Nucleus	Inhibits salicylic acid-dependent defense responses	Confocal microscopy	Enhanced jasmonic acid signaling	Transgenic *A. thaliana* expressing 35S:NPR1-GFP leaves	Confocal microscopy: 5 to 40 min	[82]
LMV	HC-Pro	20S α5	*A. thaliana*	HC-Pro aggregates	Reduces RNase activity on viral RNA	Subcellular fractionation	Protein–protein	*Lactuca sativa* leaves	BiFC: 4 dpi	[83,84]
PVX	CP, TGBp1, or TGBp2	MPB2Cb	*N. benthamiana*	ER	Blocks formation of replication compartments	Yeast two hybrid	Protein–protein	*N. benthamiana* leaves	Confocal microscopy: 2 dpi	[85]
**Nuclear proteins**										
CIRV	p36 and p95^pol^	RH30	*N. benthamiana* and *A. thaliana*	Mitochondria	Blocks assembly of the sites of replication	Confocal microscopy	Protein–protein	*N. benthamiana* leaves	Confocal microscopy: 84 h	[86]
CNV	p33 and p92^pol^	RH30	*N. benthamiana* and *A. thaliana*	Peroxisome	Blocks assembly of the sites of replication	Confocal microscopy	Protein–protein	*N. benthamiana* leaves	Confocal microscopy: 84 h	[86]
TBSV	p33 and p92^pol^	RH30	*N. benthamiana* and *A. thaliana*	Peroxisome	Blocks assembly of the sites of replication	Confocal microscopy	Protein–protein	*N. benthamiana* leaves	Confocal microscopy: 84 h	[86]
TBSV	p19	ALY1 ALY3	*A. thaliana*	Cytoplasm	Unknown	Yeast two hybrid	Protein–protein	*N. benthamiana* leaves	Confocal microscopy: 3 dpi	[79]
**Vacuolar proteins**										
CaMV	p4	NBR1	*A. thaliana*	Inclusion bodies	NBR1-dependent degradation of p4	Yeast two hybrid	Protein–protein	*N. benthamiana* leaves	Confocal microscopy: 2 dpi	[82]
TuMV	HC-Pro	NBR1	*A. thaliana*	Granule-like cytoplasmic structures	NBR1-dependent degradation of HC-Pro	Confocal microscopy	Protein–protein	Transgenic *A. thaliana* expressing NBR1-RFP leaves	Confocal microscopy on systemically infected leaves: 14 dpi	[87]

* Experimental plants are wild type unless noted.
